# Ethyl (*E*)-2-cyano-3-(4-methyl­phen­yl)acrylate: a second monoclinic polymorph

**DOI:** 10.1107/S1600536813009550

**Published:** 2013-04-17

**Authors:** Qi-Yu Chen, Wen-Dong Ke, Lin Kong

**Affiliations:** a Deparment of Chemistry, Anhui University, Hefei 230039, People’s Republic of China; b Key Laboratory of Functional Inorganic Materials, Chemistry, Hefei 230039, People’s Republic of China

## Abstract

The title compound, C_13_H_13_NO_2_, was previously described in space group *P*2_1_/*c* by He *et al.* [*Acta Cryst.* (1993), C**49**, 2000–2002]. The ethyl group is disordered over two sets of sites in a 0.615 (10):0.385 (10) ratio. The C—O—C—C torsion angles containing the ethyl group are −111.6 (10) and 177.9 (7)°, while in the previously reported polymorph, the torsion angle is −167.3 (2)°. The molecules pack to form a three-dimensional structure in the *ABAB* style along the *c*-axis direction in the title compound, but parallel to the *a*-axis direction in the reported polymorph.

## Related literature

For the first polymorph, see: He *et al.* (1993 ▶). For background to intra­molecular charge-transfer mol­ecules and their use in the construction of one- to three-dimesional organic nanostructures, see: Zhang *et al.* (2007 ▶); Zhang *et al.* (2008 ▶).
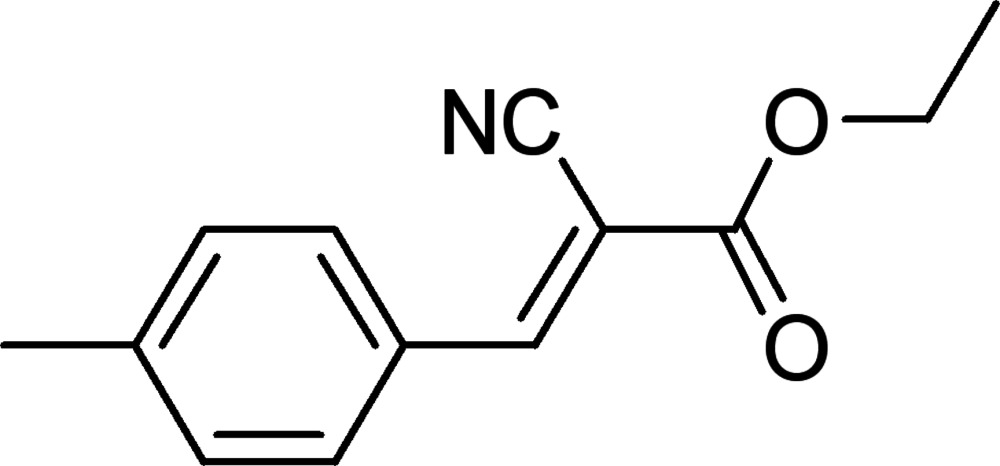


## Experimental

### 

#### Crystal data

C_13_H_13_NO_2_
*M* *_r_* = 215.24
Monoclinic, 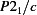

*a* = 4.7616 (4) Å
*b* = 17.7989 (15) Å
*c* = 14.2841 (12) Å
β = 93.8021 (10)°
*V* = 1207.93 (18) Å^3^
*Z* = 4
Mo *K*α radiation
μ = 0.08 mm^−1^
*T* = 298 K
0.20 × 0.20 × 0.20 mm

#### Data collection

Bruker APEXII CCD diffractometer
Absorption correction: multi-scan phi and omega scans *T*_min_ = 0.984, *T*_max_ = 0.984
8359 measured reflections
2117 independent reflections
1617 reflections with *I* > 2σ(*I*)
*R*_int_ = 0.020

#### Refinement

*R*[*F* ^2^ > 2σ(*F* ^2^)] = 0.055
*wR*(*F* ^2^) = 0.192
*S* = 1.11
2117 reflections
168 parameters
H-atom parameters constrained
Δρ_max_ = 0.19 e Å^−3^
Δρ_min_ = −0.21 e Å^−3^

### 

Data collection: *APEX2* (Bruker, 2002 ▶); cell refinement: *SAINT* (Bruker, 2002 ▶); data reduction: *SAINT*; program(s) used to solve structure: *SHELXS97* (Sheldrick, 2008 ▶); program(s) used to refine structure: *SHELXL97* (Sheldrick, 2008 ▶); molecular graphics: *SHELXTL* (Sheldrick, 2008 ▶); software used to prepare material for publication: *SHELXTL*.

## Supplementary Material

Crystal structure: contains datablock(s) I, global. DOI: 10.1107/S1600536813009550/aa2081sup1.cif

Supplementary material file. DOI: 10.1107/S1600536813009550/aa2081Isup2.mol

Structure factors: contains datablock(s) I. DOI: 10.1107/S1600536813009550/aa2081Isup3.hkl

Supplementary material file. DOI: 10.1107/S1600536813009550/aa2081Isup4.cml

Additional supplementary materials:  crystallographic information; 3D view; checkCIF report

## Figures and Tables

**Fig. 1.**
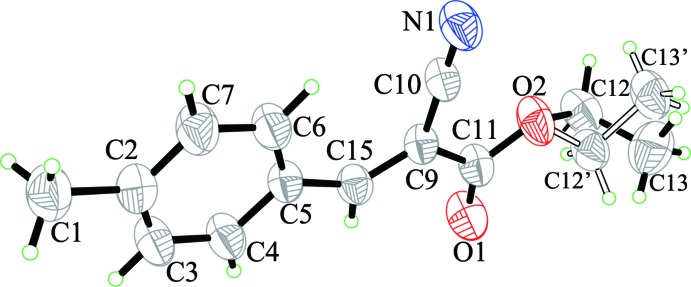
: The molecular structure of the title molecule with 50% probability ellipsoids.

## supplementary crystallographic information

### Comment

The title compound is a typical D–π–A (D = donor, A = acceptor) molecule. This type of compounds can be regarded as intramolecule charge transfer (ICT) molecules (Zhang *et al.*, 2007) and can be used to construct 1-dimesional to 3-dimesional organic nanostructures (Zhang *et al.*, 2008). In the title compound, the benzene cycle can be used as the electron donor unit, cyano group as the electron acceptor unit, the ester group as a flexible chain to increase the solubility of the compound. Thus, the cyano group and benzene cycle are linked by a vinyl bond to form organo-soluble D–π–A molecule.

In title compound (Fig. 1), the C═C group is almost coplanar with the attached phenyl ring, the torsion angle C9—C15—C5—C4 being 2.2 (4) °. The C═C group is also coplanar with ester group. The torsion angle C15—C9—Cl1—O2 is 177.5 (2) °. Excellent coplanarity of conjugated moieties enables the title compound to be a high delocalized electron system.

### Experimental

*p*-Toluic aldehyde (0.50 g), ethyl cyanoacetate(0.51 g), and ammonium acetate (0.32 g) were dissolved in 30 ml of ethanol and refluxed for about 4 h to give crude product as a solid. The precipitation was filtered, purified by recrystallization from acetonitrile/water (1:4) and 0.85 g of the titled compound was obtained as a white solid. Yield: 94.9%. ^1^H NMR (400 MHz, d^6^-DMSO): 8.35 (s, 1H), 7.98 (d, J = 8.0 Hz, 2H), 7.41 (d, J = 8.0 Hz, 2H), 7.32 (q, J = 7.2 Hz, 2H), 2,40 (s, 3H), 1.31 (t, J = 7.2 Hz, 3H). ^13^C NMR (100 MHz) 13.97, 21.33, 62.24, 101.14, 115.78, 128.68, 129.94, 130.96, 144.39, 154.94, 161.98.

### Refinement

All hydrogen atoms were placed in geometrically idealized positions and constrained to ride on their parent atoms, with C—H = 0.93—0.97 Å and *U*_iso_(H) = 1.2 or 1.5 *U*_eq_(C).

### Crystal data

**Table:**

C_13_H_13_NO_2_	*F*(000) = 456
*M**_r_* = 215.24	*D*_x_ = 1.184 Mg m^−^^3^
Monoclinic, *P*2_1_/*c*	Mo *K*α radiation, λ = 0.71073 Å
Hall symbol: -P 2ybc	Cell parameters from 3950 reflections
*a* = 4.7616 (4) Å	θ = 2.3–25.5°
*b* = 17.7989 (15) Å	µ = 0.08 mm^−^^1^
*c* = 14.2841 (12) Å	*T* = 298 K
β = 93.8021 (10)°	Needle, white
*V* = 1207.93 (18) Å^3^	0.20 × 0.20 × 0.20 mm
*Z* = 4	

### Data collection

**Table:**

Bruker APEXII CCD diffractometer	2117 independent reflections
Radiation source: fine-focus sealed tube	1617 reflections with *I* > 2σ(*I*)
Graphite monochromator	*R*_int_ = 0.020
φ and ω scans	θ_max_ = 25.0°, θ_min_ = 1.8°
Absorption correction: multi-scan phi and omega scans	*h* = −5→5
*T*_min_ = 0.984, *T*_max_ = 0.984	*k* = −21→19
8359 measured reflections	*l* = −16→16

### Refinement

**Table:**

Refinement on *F*^2^	Secondary atom site location: difference Fourier map
Least-squares matrix: full	Hydrogen site location: inferred from neighbouring sites
*R*[*F*^2^ > 2σ(*F*^2^)] = 0.055	H-atom parameters constrained
*wR*(*F*^2^) = 0.192	*w* = 1/[σ^2^(*F*_o_^2^) + (0.1011*P*)^2^ + 0.1786*P*] where *P* = (*F*_o_^2^ + 2*F*_c_^2^)/3
*S* = 1.11	(Δ/σ)_max_ < 0.001
2117 reflections	Δρ_max_ = 0.19 e Å^−^^3^
168 parameters	Δρ_min_ = −0.21 e Å^−^^3^
0 restraints	Extinction correction: *SHELXL97* (Sheldrick, 2008), Fc^*^=kFc[1+0.001xFc^2^λ^3^/sin(2θ)]^-1/4^
Primary atom site location: structure-invariant direct methods	Extinction coefficient: 0.043 (10)

### Special details

**Table:**

Geometry. All e.s.d.'s (except the e.s.d. in the dihedral angle between two l.s. planes) are estimated using the full covariance matrix. The cell e.s.d.'s are taken into account individually in the estimation of e.s.d.'s in distances, angles and torsion angles; correlations between e.s.d.'s in cell parameters are only used when they are defined by crystal symmetry. An approximate (isotropic) treatment of cell e.s.d.'s is used for estimating e.s.d.'s involving l.s. planes.
Refinement. Refinement of *F*^2^ against ALL reflections. The weighted *R*-factor *wR* and goodness of fit *S* are based on *F*^2^, conventional *R*-factors *R* are based on *F*, with *F* set to zero for negative *F*^2^. The threshold expression of *F*^2^ > σ(*F*^2^) is used only for calculating *R*-factors(gt) *etc*. and is not relevant to the choice of reflections for refinement. *R*-factors based on *F*^2^ are statistically about twice as large as those based on *F*, and *R*- factors based on ALL data will be even larger.

### Fractional atomic coordinates and isotropic or equivalent isotropic displacement parameters (Å^2^)

**Table:**

	*x*	*y*	*z*	*U*_iso_*/*U*_eq_	Occ. (<1)
C1	0.2687 (8)	−0.0915 (2)	0.4400 (2)	0.1201 (10)	
H1A	0.1208	−0.1263	0.4219	0.180*	
H1B	0.2004	−0.0549	0.4823	0.180*	
H1C	0.4242	−0.1182	0.4705	0.180*	
C2	0.3650 (6)	−0.05220 (16)	0.35330 (19)	0.0954 (8)	
C3	0.2589 (7)	0.01687 (16)	0.3251 (2)	0.1062 (9)	
H3	0.1250	0.0398	0.3601	0.127*	
C4	0.3451 (6)	0.05290 (14)	0.24691 (19)	0.0924 (8)	
H4	0.2715	0.0999	0.2307	0.111*	
C5	0.5404 (5)	0.02009 (12)	0.19193 (16)	0.0740 (6)	
C6	0.6416 (6)	−0.05037 (14)	0.21981 (19)	0.0953 (8)	
H6	0.7713	−0.0744	0.1842	0.114*	
C7	0.5541 (7)	−0.08526 (15)	0.2988 (2)	0.1009 (9)	
H7	0.6256	−0.1324	0.3153	0.121*	
C9	0.6075 (5)	0.11983 (12)	0.06603 (15)	0.0713 (6)	
C10	0.4199 (5)	0.17670 (12)	0.09614 (16)	0.0777 (7)	
C11	0.7662 (5)	0.13621 (14)	−0.01789 (17)	0.0829 (7)	
C12	0.741 (3)	0.2242 (6)	−0.1547 (6)	0.109 (3)	0.385 (10)
H12A	0.5727	0.2456	−0.1863	0.131*	0.385 (10)
H12B	0.8083	0.1828	−0.1912	0.131*	0.385 (10)
C13	0.965 (3)	0.2823 (9)	−0.1321 (9)	0.136 (4)	0.385 (10)
H13A	0.8843	0.3244	−0.1017	0.204*	0.385 (10)
H13B	1.0425	0.2987	−0.1890	0.204*	0.385 (10)
H13C	1.1117	0.2608	−0.0911	0.204*	0.385 (10)
C15	0.6509 (5)	0.05304 (12)	0.10882 (16)	0.0755 (6)	
H15	0.7755	0.0220	0.0796	0.091*	
C12'	0.8890 (16)	0.2220 (4)	−0.1310 (5)	0.0961 (19)	0.615 (10)
H12C	1.0818	0.2271	−0.1053	0.115*	0.615 (10)
H12D	0.8819	0.1840	−0.1797	0.115*	0.615 (10)
C13'	0.780 (2)	0.2953 (3)	−0.1689 (5)	0.119 (2)	0.615 (10)
H13D	0.5833	0.2904	−0.1879	0.178*	0.615 (10)
H13E	0.8814	0.3094	−0.2220	0.178*	0.615 (10)
H13F	0.8040	0.3331	−0.1212	0.178*	0.615 (10)
N1	0.2704 (6)	0.22244 (13)	0.11968 (17)	0.1036 (8)	
O1	0.9398 (5)	0.09507 (11)	−0.04727 (14)	0.1101 (7)	
O2	0.6971 (4)	0.20214 (10)	−0.05566 (13)	0.1045 (7)	

### Atomic displacement parameters (Å^2^)

**Table:**

	*U*^11^	*U*^22^	*U*^33^	*U*^12^	*U*^13^	*U*^23^
C1	0.1351 (17)	0.1184 (17)	0.1087 (15)	−0.0055 (14)	0.0237 (13)	0.0261 (13)
C2	0.1080 (15)	0.0895 (14)	0.0898 (13)	−0.0069 (12)	0.0141 (12)	0.0126 (11)
C3	0.123 (2)	0.094 (2)	0.108 (2)	0.0117 (16)	0.0474 (17)	0.0159 (15)
C4	0.1092 (18)	0.0723 (15)	0.0990 (17)	0.0114 (13)	0.0320 (14)	0.0128 (13)
C5	0.0863 (14)	0.0615 (12)	0.0751 (13)	−0.0038 (10)	0.0114 (11)	0.0015 (10)
C6	0.120 (2)	0.0713 (15)	0.0974 (18)	0.0148 (13)	0.0271 (15)	0.0122 (13)
C7	0.131 (2)	0.0749 (16)	0.0985 (18)	0.0101 (14)	0.0183 (16)	0.0203 (14)
C9	0.0841 (13)	0.0612 (12)	0.0695 (12)	−0.0036 (10)	0.0120 (10)	−0.0005 (9)
C10	0.0975 (15)	0.0600 (12)	0.0769 (14)	−0.0010 (11)	0.0151 (11)	0.0059 (10)
C11	0.1033 (17)	0.0688 (14)	0.0783 (14)	0.0001 (12)	0.0191 (12)	0.0043 (11)
C12	0.134 (8)	0.096 (6)	0.099 (6)	0.016 (6)	0.017 (5)	0.014 (4)
C13	0.137 (8)	0.144 (10)	0.132 (8)	−0.025 (8)	0.044 (7)	−0.004 (7)
C15	0.0883 (14)	0.0631 (13)	0.0762 (13)	0.0012 (10)	0.0137 (11)	−0.0020 (10)
C12'	0.104 (4)	0.097 (4)	0.090 (4)	0.011 (4)	0.031 (3)	0.026 (3)
C13'	0.151 (6)	0.101 (4)	0.109 (4)	0.011 (4)	0.042 (4)	0.037 (3)
N1	0.1314 (19)	0.0732 (14)	0.1099 (17)	0.0159 (12)	0.0344 (14)	0.0056 (12)
O1	0.1454 (17)	0.0902 (13)	0.1006 (13)	0.0211 (11)	0.0524 (12)	0.0065 (10)
O2	0.1456 (16)	0.0791 (12)	0.0937 (13)	0.0125 (10)	0.0435 (11)	0.0220 (9)

### Geometric parameters (Å, º)

**Table:**

C1—C2	1.519 (4)	C10—N1	1.147 (3)
C1—H1A	0.9600	C11—O1	1.201 (3)
C1—H1B	0.9600	C11—O2	1.324 (3)
C1—H1C	0.9600	C12—O2	1.496 (8)
C2—C7	1.363 (4)	C12—C13	1.507 (6)
C2—C3	1.379 (4)	C12—H12A	0.9700
C3—C4	1.374 (4)	C12—H12B	0.9700
C3—H3	0.9300	C13—H13A	0.9600
C4—C5	1.385 (3)	C13—H13B	0.9600
C4—H4	0.9300	C13—H13C	0.9600
C5—C6	1.392 (3)	C15—H15	0.9300
C5—C15	1.453 (3)	C12'—C13'	1.492 (5)
C6—C7	1.376 (4)	C12'—O2	1.500 (5)
C6—H6	0.9300	C12'—H12C	0.9700
C7—H7	0.9300	C12'—H12D	0.9700
C9—C15	1.346 (3)	C13'—H13D	0.9600
C9—C10	1.435 (3)	C13'—H13E	0.9600
C9—C11	1.488 (3)	C13'—H13F	0.9600
			
C2—C1—H1A	109.5	N1—C10—C9	179.5 (3)
C2—C1—H1B	109.5	O1—C11—O2	123.8 (2)
H1A—C1—H1B	109.5	O1—C11—C9	124.1 (2)
C2—C1—H1C	109.5	O2—C11—C9	112.1 (2)
H1A—C1—H1C	109.5	O2—C12—C13	96.8 (8)
H1B—C1—H1C	109.5	O2—C12—H12A	112.4
C7—C2—C3	117.4 (2)	C13—C12—H12A	112.4
C7—C2—C1	120.9 (3)	O2—C12—H12B	112.4
C3—C2—C1	121.6 (3)	C13—C12—H12B	112.4
C4—C3—C2	122.0 (3)	H12A—C12—H12B	110.0
C4—C3—H3	119.0	C9—C15—C5	132.4 (2)
C2—C3—H3	119.0	C9—C15—H15	113.8
C3—C4—C5	120.8 (2)	C5—C15—H15	113.8
C3—C4—H4	119.6	C13'—C12'—O2	104.6 (4)
C5—C4—H4	119.6	C13'—C12'—H12C	110.8
C4—C5—C6	116.7 (2)	O2—C12'—H12C	110.8
C4—C5—C15	125.9 (2)	C13'—C12'—H12D	110.8
C6—C5—C15	117.4 (2)	O2—C12'—H12D	110.8
C7—C6—C5	121.5 (2)	H12C—C12'—H12D	108.9
C7—C6—H6	119.2	C12'—C13'—H13D	109.5
C5—C6—H6	119.2	C12'—C13'—H13E	109.5
C2—C7—C6	121.4 (3)	H13D—C13'—H13E	109.5
C2—C7—H7	119.3	C12'—C13'—H13F	109.5
C6—C7—H7	119.3	H13D—C13'—H13F	109.5
C15—C9—C10	124.5 (2)	H13E—C13'—H13F	109.5
C15—C9—C11	117.9 (2)	C11—O2—C12	124.9 (6)
C10—C9—C11	117.57 (19)	C11—O2—C12'	110.8 (3)
			
C7—C2—C3—C4	−2.1 (5)	C10—C9—C11—O2	−2.9 (3)
C1—C2—C3—C4	179.9 (3)	C10—C9—C15—C5	−1.5 (4)
C2—C3—C4—C5	1.2 (5)	C11—C9—C15—C5	178.0 (2)
C3—C4—C5—C6	0.2 (4)	C4—C5—C15—C9	2.2 (4)
C3—C4—C5—C15	−178.9 (3)	C6—C5—C15—C9	−177.0 (2)
C4—C5—C6—C7	−0.7 (4)	O1—C11—O2—C12	22.4 (7)
C15—C5—C6—C7	178.5 (2)	C9—C11—O2—C12	−158.7 (6)
C3—C2—C7—C6	1.6 (5)	O1—C11—O2—C12'	−7.2 (5)
C1—C2—C7—C6	179.6 (3)	C9—C11—O2—C12'	171.8 (4)
C5—C6—C7—C2	−0.2 (5)	C13—C12—O2—C11	−111.6 (10)
C15—C9—C11—O1	−3.5 (4)	C13—C12—O2—C12'	−42.2 (9)
C10—C9—C11—O1	176.0 (2)	C13'—C12'—O2—C11	177.9 (7)
C15—C9—C11—O2	177.5 (2)	C13'—C12'—O2—C12	53.2 (10)
